# Book Reviews

**Published:** 1828-02

**Authors:** 


### CRITICAL ANALYSES.

Quæ laudanda forent, et quæ culpanda, vicissim

Illa, prius, cretâ; mox hæc, carbone, *notamus*.—PERSIUS.

*A Practical Essay on Stricture of the Rectum; illustrated by Cases, showing the Connexion of that Disease with Affections of the Urinary Organs and the Uterus, with Piles, and various Constitutional Complaints.* By FREDERICK SALMON, Surgeon to the General Dispensary, Aldersgate-street, and formerly House-Surgeon to St. Bartholomew's Hospital.—8vo. pp. 188. G. B. Whittaker, London, 1828.

THE object of this work is "to prove that stricture of the rectum is a very common disease, inducing other important affections, and that surgery furnishes us with means adequate to its removal, or alleviation, provided such means are exercised with judgment and science." (*Preface.*) Our surgical readers are no doubt well aware that it is only of late years that common stricture of the rectum has attracted much attention in this country, and that to Mr. COPELAND and Mr. WHITE, of Bath, the profession are mainly indebted for what they know upon the subject. Mr. CALVERT, indeed, latterly published his Prize Essay, which contained many valuable facts and observations; but still the profession do not appear to have hitherto attached so much importance to these diseases as they deserve,—perhaps from the repulsive nature of the means of cure, and perhaps also from a little scepticism, and something like an apprehension that the mode of cure usually recommended has been carried in some quarters to a very pernicious excess. We confess that this is the impression upon our minds; but we shall not detain our readers with our own speculations: we shall proceed at once to analyse Mr. SALMON's volume, and leave others to judge of the soundness of his views and the value of his labours. Fortunately his work is of a very manageable size, and will enable us, therefore, to comprise our remarks in a short article.

Our author, after an anatomical description of the parts involved in the disease, proceeds, in Chapter the 2d, to enumerate the Causes of Stricture of the Rectum, which may be thus enumerated:—a constitutional predisposition, arising from the narrowness of the gut at the sigmoid flexure, (which induces him to believe that it is very often a congenital disease;) any thing tending to produce local irritation in the rectum; the administration of frequent drastic purgatives; mechanical impediment from indurated fæces, and a peculiar construction of the orifice of the anus. In females there is an additional source of the disease, arising from an enlarged and tender condition of the uterus; and, finally, piles, excrescences, enlargements of the prostate gland, and fistula in ano, may all lead to this affection. Carcinoma must not be omitted among the causes. In the first instance, Mr. Salmon believes the greater portion of strictures to be merely spasmodic; but it will be easily conceived that, in process of time, depositions will form between the coats of the bowels, that it becomes thickened, wrinkled, inflamed, and ulcerated. In long protracted cases, adhesions will be formed between the rectum, uterus, and vagina; and fistulous communications will occasionally exist between them. Sometimes bands of a firm consistence are formed across the bowel.

Regarding the *situation* of stricture of the rectum, our author thinks that it takes place higher up in the gut than is usually supposed. In the greater number of cases which have fallen under his care, from five to six inches was the common situation; next in frequency, the disease occurs at the junction of the sigmoid flexure with the rectum; the very reverse of which, he thinks, is the case in carcinoma of the rectum.

We make no extracts from the third Chapter, on the Symptoms of Stricture, the detail of which differs in no way from the account usually given by other writers, but proceed to Chapter the fourth, on the Treatment of Stricture: this our author divides into constitutional and surgical. Among the first of these, indeed, we find many topical applications, together with cupping to the perineum, leeching, &c., which we should scarcely have enumerated under the head of constitutional means. Much stress is laid upon a proper management of the stomach and bowels. The local means, we are told, can never establish a recovery without a due attention to the daily regularity of the bowels. We must, in fact, take care not to overload the stomach with food, nor act upon the bowels with violent cathartics: therefore he prefers castor oil, or the senna confection, with precipitated sulphur, or injections of thin gruel with a dessert-spoonful of castor oil. Mr. Salmon employs several pages in dietetic rules and regulations: we may be excused from reciting them, not because they are prolix, but because their necessity is now very generally felt and admitted by all.

Chapter the fifth, on the Introduction of the Bougie, may be said to contain the very essence of the work; for it is by the careful and scientific use of the bougie alone that we may expect the curative measure to be accomplished, and we therefore give the following extract in the author's own words:

"One or two hours previous to the examination of the rectum, an injection is to be administered of tepid poppy water, containing forty or fifty drops of laudanum, which will tranquillise the bowel, and remove any lodgment of fæces. The patient should also be requested to make water immediately previous to the introduction of the instrument. The rectum is first to be examined with the finger, to ascertain that there is no kind of obstruction near the orifice.

"The patient, if a male, leaning over the back of the chair, or the side of a bed,* should draw aside the nates fairly to expose the orifice of the bowel. A full-sized bougie, not less than eleven inches in length, thoroughly softened and well oiled, adapted to the shape of the passage through which it is to be passed, is to be introduced with the convexity of the first curve towards the sacrum, in which way it is to be passed upwards and backwards about two inches,† through the third portion of the bowel, provided it gives no pain, for the introduction will

* "In females the examination, being made beneath the bed-clothes, is conducted without the slightest exposure.

† "The last curve of the rectum is so trifling, that it matters not whether we introduce the convex or concave part of the instrument towards the sacrum; but, by passing it with the convexity backwards, we avoid the necessity of altering the position of the bougie in passing it through the second curve of the bowel.

commonly produce an uneasy sensation; we continue to propel the bougie in the same direction, about three or three and a half inches higher, or through the second portion of the rectum; the point of the instrument will now bear directly upon the hollow of the sacrum, and the but-end towards the left side of the body. With a view, therefore, of avoiding the sacrum, and of accommodating the instrument to the great curve of the rectum, we change its position, by describing the segment of a circle from left to right, with the but-end, turning it upwards, at the same time continuing to propel the instrument. Having described this segment, we shall have carried the bougie full four inches farther, or to what may be considered the extent of the rectum. But it is yet to be introduced into the sigmoid flexure: we therefore triflingly depress the but of the instrument, at the same time propelling it upwards, till the whole is fairly within the sphincter: this accomplished, we may be satisfied.

"Having passed the instrument between five and six inches, the patient generally complains of pain not only in the rectum, but over the surface of the abdomen, particularly in the umbilical region.

"Upon encountering obstruction, trifling pressure is to be maintained for a minute or two, and if under this careful pressure the pain increase, and the instrument remain stationary, it is to be withdrawn, the next size introduced, and so on from above downwards, till we ascertain the size which passes with trifling pain or difficulty into the sigmoid flexure. Should we doubt whether the bougie has entered this part, the doubt will be removed by relinquishing the pressure from the end of the instrument, when, if the bougie has not entered the sigmoid flexure, it will slowly recoil; we are occasionally apprised of its having entered this part by the passage of air from the bowel.

"The bougie is to remain in the bowel ten or fifteen minutes, provided it produces no considerable irritation, care being taken to affix a tape through the loop to prevent the instrument being drawn up into the rectum; at the expiration of that time it is to be removed, and allowed to harden in the shape to which the intestine has moulded it: this will be of considerable service in the subsequent treatment of the case, acting as a guide by which we adapt the several instruments throughout the curative process. In withdrawing the bougie, we should bear in mind the anatomy of the rectum, being cautious not to strike the point of the instrument against the angles of the intestine.

"Very commonly the two or three first introductions of the instrument cause a distressing desire to relieve the bowels: this desire will, however, gradually subside.

"At intervals of from three to five days, the operation is to be repeated, increasing the size of the bougie, and the period it is suffered to remain in the intestine, according as the circumstances of the case permit. We shall commonly be enabled to use a larger instrument on the second, third, and fourth introductions, when that we commenced with does not exceed No. 4; but, when the size originally passed reaches No. 7, we shall seldom be enabled to increase it more frequently than at every other introduction. As the instruments become of larger size, we shall be necessitated to pass the same two, three, and frequently even four times; but no established rule can be laid down upon this point, which must depend upon the extent and nature of the contraction, equally with the constitutional irritability of the patient. (P. 40.)

The mode in which the bougie acts is twofold, by dilatation and absorption.

With regard to the quality of the bougie, our author observes that the composition recommended by Mr. WHITE has not sufficient density, and is only adapted for cases where the bowel is very irritable. The instrument used by Mr. Salmon is composed of fine linen cloth, very heavily coated with wax, and a certain proportion of diachylon plaster, mixed with a small quantity of lampblack: it is to be immersed in very hot water some minutes previously to being used, which renders it pliant without depriving it of one regular smooth surface. The largest sized bougie is three and a half inches in circumference; the smallest, one inch; the medium between the two is divided into ten sizes. The length of the bougie is about eleven inches. Our author condemns metallic rectum bougies, and disapproves of the gum-elastic one. When the stricture is accompanied with great local irritation, the bougie may be smeared over with an ointment, composed of an ounce of elder ointment, with a scruple of very finely powdered opium.

We are next instructed, in Chapter 6th, in the mode of discontinuing the use of the bougie, as well as the inconveniencies sometimes attending its employment. The latter may be thus enumerated: numbness in the thighs and legs, shiverings, pains over the whole abdomen, powerful action of the sphincter muscle, and a great liability to taking cold, or mucous irritation during the process. When the disease is situated high up, a considerable length of time is necessary for the cure. Sometimes a few introductions of the bougie will be sufficient: they should be persisted in until No. 8 can be readily passed into the sigmoid flexure; and they are to be discontinued gradually, until it is employed merely as a preventive once in six weeks or two months.

Chapter the 7th, on Dividing the Stricture, we pass by. The plan is not recommended by our author, and the mode of performing the operation is not novel. The following, Chapter the 8th, is very short: it describes the morbid anatomy, symptoms, &c. of Carcinoma of the Rectum, but contains nothing that need detain us a moment, and certainly adds nothing to what was previously known of this fatal affection.

We have now gone through the general remarks contained in Mr. Salmon's work. The remaining chapters, by far the largest portion of the volume, are occupied in describing the effects of stricture of the rectum upon contiguous or other organs; and, as they are chiefly illustrated by cases, it is obviously impossible to abridge these with any advantage to our readers, and we must therefore content ourselves with giving the heads of the chapters, or very little more.

The 9th Chapter contains remarks upon Affections of the Urinary Organs, or Kidneys, as connected with stricture of the rectum, and which may be readily comprehended from the relative situation of the parts.

The 10th Chapter on Affections of the Uterus and Vagina, contains some remarks which we must notice.

"We shall commonly find the uterus sympathise with the rectum, and indistinct appearances arise, leading us to suspect incipient disease in the former organ, which disease will totally disappear upon the removal of stricture in the rectum. I have known discharges from the vagina to subside upon the relief of stricture, and I am of opinion that many cases of miscarriage have their origin in that complaint. This appears probable, when we reflect upon the contiguity of the parts. From any accumulation in the rectum, pressure is made upon the uterus, which pressure is increased at each attempt to relieve the bowels: this naturally irritates the uterus, disturbing its functions, and giving rise, during the period of gestation more especially, to those irregular contractions which may ultimately cause it to reject its contents. (P. 102.)

These points are illustrated by the relation of several cases.

The 11th Chapter is devoted to the consideration of Distention and permanent Enlargement of the Colon, chiefly introductory to two cases of this affection, which are detailed at great length.

We must here conclude our extracts. There are yet two chapters in the book: one on Piles, the other on Determination of Blood to the Head, as a consequence of stricture; they are each followed by the relation of numerous cases.

It may be expected that we should conclude this article with something like an opinion as to the merit of our author's production. We think, then, that Mr. Salmon has compressed into a narrow compass all that can be said upon his subject. There is, perhaps, very little novelty in his doctrines; and there is some doubt in our minds with regard to the very common occurrence of the disease as insisted upon by our author; and, moreover, we are not without our fears that it may induce practitioners to look too much to this source of disease, to the exclusion of other more general and constitutional causes of the various symptoms which are here said to exist so often in connexion with stricture of the rectum.

---

*Observations on the Mortality and Physical Management of Children.* By John Roberton, Member of the Royal College of Surgeons, Edinburgh; of the Literary and Philosophical Society of Manchester; and one of the Surgeons to the Manchester Lying-in Hospital.—12mo. pp. 311. Longman and Co. London, 1827.

This Treatise is divided into two parts. Part the first is designed to show the amount as well as the causes of infantile mortality in this country. In the second part, that department only of the physical management of children is attempted, which comprehends the maintenance and improvement of the health: that which relates to the cultivation of the individual faculties has been omitted, as foreign to the design of the work. Several tables are given, showing the rate of infantile mortality in different places. Such documents would doubtless be interesting in many points of view, if their correctness could possibly be relied on. But the carelessness with which such registers are usually kept in this country, particularly as far as regards the causes of death amongst children, is too notorious to warrant us in drawing any useful conclusions from the aggregate numbers thus recorded. The proportional mortality under ten years of age, in foreign countries, nearly approaches that in our own. According to Susmilch's German Tables, it is 43 per cent. for the country, 47.7 for small towns, and 50.2 for large cities. In France, according to Duvillard, it is 44.89 on the average of the kingdom. In different places, a variety of local circumstances produces a corresponding difference in the ratio of mortality amongst children of an early age.

"When we consider the peculiar circumstances of the labouring poor in manufacturing districts, their frequent privations, their improvidence, ignorance, and vicious habits,—and the low, badly ventilated abodes of the majority of such of them as live in large towns,—we cease to wonder that so few of their children comparatively should be reared.

"In Manchester and Glasgow, many of the poorest class are Irish. It is difficult to estimate their number, but it cannot be less than twenty-five or thirty thousand in each place. Their habits are well known. In confined filthy yards, noisome cellars, old ruined buildings, we are sure to find this singular people the principal tenants, huddled together in their peculiar way, and, in spite of every privation, marvellously content. So perfectly do most of them continue to maintain their native habits in this country, that no conveniences and comforts which they see others of their own condition enjoy, and which they might as easily attain, do in the least degree stimulate them; a proof how slow and difficult is the progress from barbarism to civilised life." (P. 32.)

The following sketch of the apathy of the lower classes of the Irish will afford sufficient proof of the probable degree of mortality amongst their children. It is taken from a work on the Population of Dublin, by the Rev. J. Whitlaw. In one house this gentleman frequently found thirty or forty individuals, and, some years before the period of his survey, No. 6, Braithwaite-street, contained 108 human beings.

"This crowded population, where it obtains, is almost universally accompanied by a very serious evil: a degree of filth and stench inconceivable except by such as have visited these scenes of wretchedness. Into the back yard of each house, frequently not ten feet deep, is flung, from the windows of each apartment, the ordure and other filth of its numerous inhabitants, from whence it is so seldom removed that I have seen it nearly on a level with the windows of the first floor; and the moisture that, after heavy rains, oozes from this heap, having frequently no sewer to carry it off, runs into the street by an entry leading to the staircase. One instance out of a thousand that might be given, will be sufficient. When I attempted, in the summer of 1798, to take the population of a ruinous house in Joseph's-lane, near Castle Market, I was interrupted in my progress by an inundation of putrid flood, alive with maggots, which had, from an adjacent slaughter-yard, burst the back door, and filled the hall to the depth of several inches. By the help of a plank and some stepping stones, which I procured for that purpose, (for the inhabitants, without any concern, waded through it,) I reached the staircase. It had rained violently, and, from the shattered state of the roof, a torrent of water made its way through every floor, from the garret to the ground. The sallow looks and filth of the wretches who crowded round me indicated their situation, though they seemed insensible to the stench which I could scarce sustain for a few minutes. I counted in this sty thirty-seven persons; and computed that its humane proprietor received out of an absolute ruin, which should be taken down by the magistrates as a public nuisance, a profit rent of above £30. per annum, which he exacted every Saturday night with unfeeling severity. I will not disgust the reader with any further detail, and only observe, that I generally found room-keepers of this description, notwithstanding so many causes of wretchedness, apparently at ease, and perfectly assimilated to their habitations. Filth and stench seemed congenial to their nature; they never made the smallest effort to remove them; and, if they could answer the calls of hunger, they felt, or seemed to feel, nothing else as an inconvenience." (P. 34.)

In every community subsisting wholly by trade, a variety of unfavorable circumstances affect the children of the poor, some of which are unavoidable, and others, and by far the worst, spring from vices that are always prevalent in a crowded state of society.

"To show how rapidly large towns would become depopulated, were they not constantly recruited by migration, and how fatal they prove to human life, even in circumstances otherwise the most favorable to its preservation, Dr. Percival procured an account of the mortality occurring in the Society of Friends in Manchester during seven years. In this Society he found there were 81 males and 84 females, of whom 54 were married, 9 were widowers, 7 widows, and 48 under fifteen years of age. The births in seven years were 34, the burials 47: one person, therefore, in 24.6 died annually. The result is curious, and the reverse of what was to be expected.

"Dr. P. ascribes so high a rate of mortality, proportionally higher than that of the whole town, to the Society, during the above period, having had little or no accession to its number of members from the country. Indeed, without a constant supply from this source, there is reason to believe large towns would be depopulated at even a speedier rate than in the instance just given." (P. 40.)

It has been shown by various respectable authorities that infantile diseases in general, and especially measles, have been more fatal since the cow-pox was substituted for small-pox. But, granting this fact, the inference that has been drawn from it, to the prejudice of vaccination, is neither logical nor correct. Immense numbers have been snatched from the extensive ravages of small-pox, and the victims to other maladies must necessarily be increased from the greater numbers of children affected by them. Dr. Watt, of Glasgow, indeed imagines that, when the small-pox was in full force, it had the power of modifying measles, and rendering them mild, but that now, as small-pox is nearly extinct, measles are become as dangerous as the former used to be. Whether erroneous or not, these sentiments of Dr. Watts are said by Dr. Roberton to have impeded the progress of vaccination in that country, and it is therefore of much consequence to expose their fallacy.

"Recurring to the fact, which the tables prove, that more die of measles now than died formerly, and that this change appears to be an effect of vaccination, does it follow that measles are in their nature become more severe and fatal? Dr. Watt, Sir G.

Blane, and Mr. Milne agree in the affirmative : the proofs they bring forward, however, are by no means satisfactory.

"It is not enough to show that more die of measles at present, than died previously to the vaccine period ; a consequence to be expected, owing to the diminished mortality from small-pox. It should also be shown that, of a given number of cases of measles, a greater proportion dies now than died any time "when small-pox was in full force." Facts of this kind are not adduced, and had there been such in the possession of these gentlemen we may presume they would not have been withheld.

"The real cause of the increased mortality from measles and other infantile complaints may receive elucidation from the following considerations. Small-pox, owing probably to the greater abundance and virulence of its effluvia, was generally caught in a casual way, before measles and other infectious complaints, and swept off the more feeble and sickly, leaving the strong and vigorous only to encounter the attacks of other diseases." (P. 55 )

In the second part of the volume, we have a slight sketch of the physical management of children. The various opinions stated by the author have been repeated over and over again by different writers.. Their general accuracy we do not dispute, but the necessity for their republication we must be allowed to doubt. Mr. Roberton promises at a future period to offer some account of the diseases of children: we trust we shall be justified in passing a more favorable verdict on his second production, than we can conscientiously on that we have just noticed.

---

***Diseases of the Bronchia; being the first Part of a Treatise on the Diseases of the Chest and on Mediate Auscultation.*** By R. T. H. Laennec, m.d. Regius Professor of Medicine in the College of France, Clinical Professor to the Faculty of Medicine of Paris, Physician to her Royal Highness the Duchess of Berri, &c. &c. &c. *Second Edition, greatly enlarged: translated from the French, with Notes and a Sketch of the Author's Life,* by John Forbes, m.d. Member of the Royal College of Physicians, and senior Physician to the Chichester Infirmary. With Plates.—8vo. pp. 708. T. and G. Underwood, London, 1827.

The first part of Laennec's work treats of the diseases of the Bronchia, Lungs, and Pleura ; the second, of the Heart and its appendages. In speaking of these, no attempt is made to subdivide them into genera and species ; still less is any endeavour made to ascertain what has been so absurdly called the proximate cause of disease. His object is to state the characters by which diseases may be recognised during life, —the traces which they leave after death,—and the treatment which multiplied experience has shown to be efficient.

When the disease is dependent upon change of structure, the description begins with this organic alteration.

The various lesions to which the textures of the lungs are liable are first noticed, and afterwards those diseases of function in which no change of structure can be detected, and which are therefore regarded as nervous affections.

The first book relates to diseases of the Bronchia, and the first chapter of this to their inflammatory affections.

Inflammation of the bronchia is divided into the *catarrhal*, the *plastic* or *crusty*, and the *ulcerous*. Pulmonary catarrh is again subdivided into, 1, the acute mucous; 2, the chronic mucous; 3, the pituitous; 4, the dry. The term catarrh is preferred to that of bronchitis, because it forms a link which unites inflammation to congestion, and because it is sometimes doubtful whether the disease be really an inflammation or not. Catarrh is very frequent; indeed, most persons are affected with it almost every year; yet LAENNEC thinks its nature imperfectly understood. Sometimes it resembles croup, but more frequently it exhibits the characters of a simple congestion. Its anatomical peculiarities consist in some degree of redness and slight thickening of the inner membrane of the bronchia; and these appearances very rarely occupy the whole even of one lung. The extent and intensity of the redness are not always in proportion to the severity of the attack. Thus, in the catarrh which complicates fevers, the membrane is of a livid red over almost its whole extent; while, in the idiopathic disease, it shows marks of inflammation only in certain points, even when very acute. The pulmonary catarrh is attended with a change of the bronchial secretion, which becomes scantier than natural, and is sometimes almost entirely suppressed. It next becomes thin, transparent, and acrid; then, without losing its transparency, it is more viscid, like the white of an egg. After this it assumes a yellowish or greenish colour, being at the same time more consistent; in which state it obstructs the air-tubes, giving rise to the mucous rattle. By the different characters of the expectoration in the successive stages are recognised the principal varieties of the disease: thus, the dry catarrh never proceeds beyond the first stage; the pituitous stops at the second; and the mucous persists in the third.

The symptoms are too well known to require description, and we shall only allude to those which are pointed out by Laennec as pathognomonic. When the catarrh is simple, however, violent percussion every where gives the natural sound. At the invasion of the disease, while there is as yet but little cough, the stethoscope indicates a rattle, which is often loud, and may sometimes be heard at a considerable distance from its site; it is usually sonorous and deep, but sometimes sibilous. As the disease advances, the rattle becomes mucous. If the disease be partial, the rattle also is partial; but, if this be heard over the whole of one lung, or the greater part of both, the disease is always severe; and, when it exists over the whole of both, death almost invariably follows.

Except in severe cases, little in the way of medical treatment is required. Even when the attack is acute, Laennec does not recommend blood-letting, except by leeches or cupping. Blisters he thinks injurious, except when the complaint threatens to become chronic. Our author alludes to the popular plan of treating catarrh with stimulating drinks, such as warm negus or punch, a method which he states to be eminently successful in a vast number of cases, and that he always employs this kind of treatment where there exists no contra-indications, such as inflammation of the stomach or bowels, a constitution easily excited by spirits, or an attack so violent as to threaten to pass into croup. His plan is to give an ounce or an ounce and a half of brandy in infusion of violets, made hot, and sweetened with syrup of marsh-mallows; which appears to us to be nothing more than prescribing a tumbler of warm brandy punch.

The anatomical characters of the chronic catarrh are extremely similar to those of the acute, and the expectoration of the former is precisely similar to that of the advanced stage of the latter: the quantity, however, is almost always greater than in the acute disease, occasionally amounting to one or two pounds in twenty-four hours. The treatment most approved of by our author, especially in old persons, is the establishment of a permanent drain in the arm, or thigh, with the use of certain aromatics, squill, and paregorics. Our author also speaks favorably of emetics, repeated according to the patient's strength and power of supporting them. We can ourselves bear testimony to this point from considerable experience. When the lungs are oppressed by an excessive accumulation of viscid mucus, and the expectoration very difficult, we know of no remedy capable of affording so much or such speedy relief. For this purpose the sulphate of zinc has appeared to us the most efficacious medicine: it generally requires to be given in doses of a scruple or half a drachm; and, when free vomiting has been produced, the patient frequently passes a good night, and remains for a day or two comparatively free from complaint. After this the symptoms gradually return, and the remedy requires to be repeated.

"In the case of an old lady of eighty-five," says Laennec, "who had laboured under chronic catarrh for eighteen months, with an expectoration amounting to two pounds daily, I prescribed fifteen emetics in one month, and with complete success, as the patient lived eight years afterwards free from the complaint."

The author likewise speaks favorably of the balsams of tolu and copaiba, and of turpentine.

The name of *pituitous* catarrh is given to that variety of the disease in which the expectoration is "colourless, transparent, ropy, frothy on the surface, and underneath like white of egg diluted with water." It is considered by our author as intermediate between the serous and sanguineous congestions, exhibiting a middling degree of swelling, and slight softening of the mucous membrane, with some redness here and there. The acute and chronic forms are thus described :

"The *acute pituitous catarrh* constitutes one of the severest species of the suffocative catarrh. It is characterised by an extreme oppression, attended by a copious pituitous expectoration. It sometimes begins as a common cold ; but after a few hours, or even minutes, its severe character is soon declared by the violence of the cough, the intensity of the dyspnœa and oppression, the lividity of the face, marks of cerebral congestion, disordered circulation, and coldness of the extremities. In children it is sometimes mistaken for croup. I had occasion to know a case of this kind lately, where, on examining the body after death, we found the bronchia hardly at all red, but nearly filled with a serous fluid, which was somewhat viscid and slightly frothy.

"The stethoscopic signs of this affection are the varieties of rattle already noticed. To these may be added the occasional presence of a crepitous rattle, produced by a certain degree of œdema of the lungs co-existing with the serous discharge. The chest sounds well on percussion.

"These attacks, however violent, are usually transient. In certain cases they are recurrent. A remarkable instance of this is given by Bree, of a woman attacked in perfect health with a paroxysm of the kind described, passing entirely off after a few hours, and returning in the same manner and with extreme violence, after an interval of six months. In the second attack, this patient expectorated four pints of a frothy serum slightly tinged with blood. I shall notice the treatment of this complaint when I come to treat of the suffocative catarrh.

"The *chronic pituitous catarrh* occurs only in advanced life, and more particularly attacks those whose constitutions have been debilitated by excesses, or by sedentary habits. It is common in gouty subjects in whom the gout has lost its regular form, and become less strongly marked. It is also the consequence of frequent attacks of the acute mucous catarrh. The chronic variety never succeeds the acute species which we have just described. It usually comes on by slow degrees after repeated attacks of the acute mucous or dry catarrh. When the pituitous discharge is once fully established, it becomes frequently intermittent, and often with considerable regularity. There are usually two paroxysms of cough and expectoration in the twenty-four hours,—the one on waking from sleep, the other in the evening; but sometimes the paroxysms immediately follow the patient's meals. The quantity of fluid expectorated is always very great: I have known some patients discharge, in the course of one or two hours, from two to three pounds.

"During the attack there is always dyspnœa, which either diminishes or passes off with it. When this disease has existed some time, the countenance assumes a pale blueish tint, and the body becomes considerably but not extremely emaciated. The patient's constitution becomes more lymphatic; the blood grows thinner, and, when drawn from the veins, exhibits a very weak coagulum. The patient nevertheless continues fit for many avocations, and can be considered only as an invalid. In this state the complaint may exist a great many years, but, as age advances, the fits become longer and more frequent, the dyspnœa becomes habitual, and the disorder then acquires the name of *asthma.* The usual termination of it is by the supervention of œdema of the lungs, and finally suffocation from inability to expectorate.

"It is singular how many years patients will survive under the immense discharge produced by this disorder. M. Alard mentions some interesting examples of this kind; and I myself am acquainted with two old gentlemen, whose cases may be added to the number. One of these, who is upwards of seventy, has expectorated during the last ten or twelve years, in two daily paroxysms, about four pounds of a colourless ropy and frothy fluid. The other brings up every morning, by gentle spontaneous vomitings, repeated at short intervals during several hours, from three to six pounds of a liquid exactly like the white of egg mixed with a third part of water. This gentleman is upwards of sixty, enjoys tolerable health, and walks several hours every day. Some patients, however, die of exhaustion within a much shorter period, and from a much smaller discharge. Two cases of this kind are given by M. Andral:—the one, an old man, carried off after five months, by an expectoration of about two pints of a serous fluid, daily;—and the other, a person of forty-five years of age, who died after bringing up three pints of the same kind during three successive years. In neither of these cases was there found any other cause of death; and, in the one last mentioned, the mucous membrane of the bronchia was found extremely pale: so true it is that, besides the light, no doubt very great, thrown by morbid anatomy on the causes of these diseases, we must, in certain cases, look elsewhere for information.

"It is rare that we meet with idiopathic cases of this disorder so well marked as those just noticed; but it often exists in a high degree as a consequence of the simultaneous development and persistence of a great number of miliary tubercles in the lungs. These pituitous discharges were indeed considered by Bayle as the pathognomonic sign of this variety of phthisis. The symptomatic pituitous discharges are marked by less regular fits of coughing, and they present, moreover, particular signs, depending on their organic cause, which will be noticed when we come to treat of phthisis." (P. 80.)

When this disease has become habitual, it is but little under the control of medicine. The same remedies, however, may be tried which we have mentioned as applicable to the preceding variety.

The *suffocative* catarrh of Laennec seems to consist rather in the termination of the several species, from interruption to the function of respiration, rather than in any thing which can be looked upon as a distinct disease.

The *dry* catarrh is that which is attended with little or no expectoration. In the acute state, it is only one stage of a common cold: in the chronic form, it is extremely common. The anatomical characters are swelling of the mucous membrane, with redness of a violet hue. The minute branches are frequently blocked up by a very glutinous secretion, disposed in globules, which are of a pearl grey colour. The pathognomonic signs of this affection are perfect resonance of the chest, and a want of the natural sound in the parts actually affected. The respiration continues natural in the parts of the lungs which remain sound, but frequent changes take place, so that the part which at first gave no respiratory sound may, after a short time, give it very distinctly, and vice versâ. The symptoms are thus described:

"This disease, when existing in a middling degree, frequently remains altogether latent for a long course of years, the subjects of it being no further conscious of its presence than by observing that they are shorter breathed than others, when they ascend an elevation or attempt to run. When the bronchial tumefaction becomes more extended, dyspnœa is then experienced, even in a state of quietude, and particularly after meals; and this state of oppression is referred by some patients to one side only, and sometimes to the side least affected. After a time, the dyspnœa comes on in fits, which last usually several days, and are so severe as to merit and obtain the name of asthma. Towards the termination of these attacks, a cough comes on, and the oppression becomes less; and, after the cough has continued a few days, the dyspnœa is still further relieved by the expectoration of some of the pearly sputa, intermixed with phlegm. In the slighter cases, these sputa lose their usual consistence and globular form, and become more copious, and feebly opaline from an intimate intermixture of a small quantity of an opaque yellowish or whitish mucus. At other times they are vitriform, and nearly of the consistence of the vitreous humor of the eye, constituting, no doubt, the glassy pituita of the ancients. An expectoration of this kind is habitual with many persons affected with a slight degree of the dry catarrh; and, as long as it continues, they are never liable to attacks of asthma. Frequently this expectoration is in such small quantity that the patients are themselves unconscious either of it or the cough; in some persons there is, in fact, neither the one nor the other; and in many there is merely a slight cough, perfectly dry, and perceptible only once daily, or once in two or three days. Coughs of this kind, when the dry catarrh of which they are the symptom has come on slowly and without being preceded by an acute affection, are usually denominated nervous. Too frequently, indeed, they are considered sympathetic, and the cause of them is sought for in some real or supposed affection of the stomach, liver, kidneys, or uterus. Hence the coughs called gastric, hepatic, hysteric, &c.; all of which are, in fact, examples of the coexistence of the dry catarrh with some affection of the particular organs indicated. Very commonly the cough ceases entirely during summer, and the oppression becomes less: no doubt because the increase of the cutaneous transpiration diminishes the gorged state of the bronchia and the secretion of the pearly sputa.

" When a person subject to an habitual dry catarrh is attacked with an acute catarrh, this rarely goes through its regular course, so as to give rise to the copious mucous expectoration characteristic of it. On the contrary, it seems never to get beyond the first stage; but after a few days the cough becomes more frequent, and is attended by a slight pituitous expectoration, and a greater discharge than usual of pearly sputa of a thinner quality. Sometimes, indeed, their consistence is so much diminished that they lose their rounded form and become diffluent; and in this state they exhibit a compound of the pearly and glassy sputa, and the yellow and viscid mucus of common catarrh, rendered opaque and greyish by the intermixture of much black pulmonary matter. The supervention of the acute catarrh usually brings on a fit of asthma, or at least aggravates the habitual dyspnœa. This is relieved by the appearance of the expectoration; but it frequently still continues worse than before the invasion of the new affection. If fever comes on in the course of the acute catarrh, it perceptibly lessens the oppression. The same is true of sleep; and, if this ever comes on during an asthmatic paroxysm, the moment of awaking is the only one in which the patient fancies that he can breathe freely. Nevertheless, even during these intervals of ease, the respiration, examined by the stethoscope, is not at all more perfect than during the severest paroxysms; a fact which proves that both fever and sleep act in this case by diminishing the necessity of respiration. The upright posture is not so constantly requisite in cases of asthma depending on the dry catarrh, as in oppressed breathing produced by diseases of the heart or effusions into the chest. After an extensive dry catarrh has continued for a certain length of time, and more particularly if aggravated by repeated attacks of the acute kind above described, emphysema of the lungs supervenes, with its characteristic signs and symptoms. The name of *neglected cold*, usually given to phthisis, is therefore much more applicable to this latter affection.

"The dry catarrh is denominated *latent* when it is unattended either by cough or expectoration. This variety is distinguished by a very great, and usually unequal, feebleness of the respiratory sound over the greater part of the chest; by the complete sonorousness of this cavity on percussion; and by the occasional, though rare, addition of a slight sibilous or obscure mucous rattle, or very feeble sound of the valve: these last signs are of such infrequent occurrence, that we may explore the chest several days successively without perceiving them. The dry catarrh is almost always latent, particularly when slight and of small extent. It only then becomes an inconvenience when the tumefaction of the bronchia has increased sufficiently, either in extent or intensity, to impede the full development of the lungs required during exercise.

"The symptomatic catarrh of fevers is almost always latent, particularly during the first days of the disease: not but that the patients, in this case, cough occasionally; but the cough is so slight and infrequent as to be either unattended to or altogether overlooked by the physician. It is sometimes, however, noticed by the attendants, when unobserved by the practitioner. After continued fevers, and mucous catarrhs of long standing or frequently renewed, habitual latent dry catarrhs are left, which eventually terminate in the production of asthma and emphysema of the lungs.

"The frequency of the dry catarrh, the insidious slowness of its progress, and the severity of its effects when arrived at its height, ought to convince us how necessary it is not to consider as unimportant affections dry coughs of long standing, however slight or infrequent they may be. These coughs, as I have already said, are the consequence of the dry catarrh, except in the particular case wherein they are caused by the development of miliary tubercles in the lungs." (P. 89.)

The remedies which are most useful in the other forms of catarrh, are unavailing in this. The indications are to relieve the vascular congestion, and to promote expectoration: with regard to the first, blood-letting is regarded by Laennec as useless, while blisters, emetics, and purgatives, afford but temporary relief; the second indication, according to our author, is best fulfilled by the use of alkalies; on which subject he thus expresses himself:—

"As to the second indication, the removal of the pearly sputa, it is evident that the glutinous tenacity of these is the chief obstacle to their ready expulsion; and I am of opinion that we possess means, if not infallible, at least often efficacious, in lessening this tenacity of the secretions, and rendering them more liquid. This practice may perhaps seem to rest on the exploded humoral pathology, and I must confess that the theory of it is neither mine nor of this age. Sarcone and Morgagni, after many others, made this theory one of the bases of their practice. I attach no value to it as a theory; but I can state from experience that, by means of those medicines which the chemical and humoral physicians of the last three centuries considered as proper to correct the tenacity of the fluids, I have succeeded in procuring very great and permanent relief to many individuals who had long laboured under dry catarrhs of great severity. The means employed with this view are chiefly the milder or very dilute alkalies. Those I have been in the habit of using are the following:—1. Almond soap, taken in the form of pill with the patient's meals, to the amount of from half a drachm to a drachm daily. If the catarrh is complicated with spasm of the bronchia (to be noticed hereafter), I sometimes conjoin with the pills the gum ammoniac, in the dose of from eight to twenty-four grains in the day. 2. The salt-water bath, of the temperature of from ninety-three to ninety-nine degrees of Fahrenheit; the artificial alkaline bath, with four ounces of the carbonate of potass or soda; and the sulphur baths, natural or artificial. I give the preference to the last in herpetic cases. 3. The internal use of the carbonates of soda, potass, or ammonia, in a dose of from twelve to thirty-six grains per day, diffused in all the patient's drinks; or the sulphureous saline mineral waters, particularly of Bonnes and Cauterets.

"The employment of these means ought to be persevered in for several months at least, even when they afford the most speedy relief. I have never observed any ill effects from them, and I have frequently employed the soap, more especially, for two or three years without intermission. A great many persons, who had already emphysema of the lungs, and either incessant dyspnœa or very frequent fits of asthma, have, to my own knowledge, been restored, under this kind of treatment, to a state of health so comfortable, that they hardly exhibited any signs of disease, and considered themselves as entirely cured. After the employment of these means for a certain period, the pearly sputa become more abundant; or if there had been previously no expectoration of the sort, this now takes place. At the same time the tenacity of the sputa is diminished; they become diffluent, and lose their rounded form; and relief of the oppression is experienced. This plan of treatment is often most efficacious where the disease is most severe. I know not what may be thought of the theory on which it is founded. Animal chemistry is yet too imperfect to furnish the solution of the problem. No doubt it would be better if we could dispense with all theory; but this is impossible: the numerous and diverse facts which constitute the science of physic can only be classed in the memory by the aid of some systematic bond. It is, indeed, much to be desired that less importance were attributed to views, which, after all, can only be considered as the scaffolding of the science; and more especially is it to be wished that the attachment to theory would not lead many persons (as it does) to reject the very facts on which other theories, whether ancient or modern, hostile to their own, are founded." (P. 92.)

*Convulsive* catarrh, or hooping cough, is next spoken of. Stethoscopic exploration affords only the usual results of common catarrh. The peculiar sonorous respiration depends entirely upon the larynx and neighbouring parts. With regard to the treatment, it is observed that, if the patient can be made to drink by small and repeated portions during the paroxysm, this is diminished in violence, the effects of deglutition probably counteracting the spasm. As an emetic, Laennec prefers tartarised antimony to ipecacuanha; and, of the narcotics, he thinks belladonna the best. The dose is from an eighth to half a grain. When the paroxysms become periodical, bark or quina are recommended. He has seldom found blisters of much use.

*Symptomatic* catarrhs are those which habitually coexist with various other diseases. It is asserted, for example, that a catarrhal affection, either latent or manifest, exists during the whole course of continued fevers: in the exanthemata, the catarrh is equally constant, and more manifest; and even during the paroxysms of ague, symptoms of catarrh, it is said, may be detected by the stethoscope.

*Dilatation of the Bronchia* is the organic lesion next described. Sometimes this exists in one or many branches, without any other appearance of disease, and then dilated portions frequently arise from a trunk much smaller than themselves: occasionally they resume their natural size, but more frequently they terminate in a cul de sac. When this lesion is confined to the smaller branches, it is very easily overlooked in dissection. Dilatation of the bronchia has many symptoms in common with other diseases, particularly phthisis, and, although Laennec asserts that an experienced practitioner can never have any difficulty in the diagnosis, yet it is acknowledged by his ingenious translator, as well as by M. Louis, that it is extremely difficult to distinguish them. For this reason we shall say nothing of the treatment.

The next chapter is devoted to the consideration of *Croup*, and the only part of it to which we shall allude is that which relates to the treatment.

"If croup is not accompanied by a strongly marked asthenic diathesis, or does not occur in very young infants, the treatment ought to commence with one or two bleedings from the arm or foot. In doubtful cases, it would seem preferable rather to omit bleeding than to destroy, by injudicious depletion, the powers requisite for the separation and excretion of the false membrane. Blood-letting, indeed, in this, as in all other diseases which have reached the period of suppuration, is rather a preventive of future mischief than a measure likely to lessen that which already exists. And, in fact, in the case in question, the danger arises much less from the inflammation than from the mechanical obstacle to the respiration occasioned by the false membrane. In children, leeches to the throat, repeated more or less frequently according to the strength of the patient and the severity of the disease, may advantageously take the place of venesection; and, in adults, their repeated application may be useful after general bleeding has been had recourse to. Leeches have the advantage of producing, in addition to the unloading of the capillaries in the vicinity of the affected part, a sort of local eruption, which unquestionably is sometimes beneficial as a derivative. Other derivatives, however, and of the most energetic kind, must be put in requisition, particularly blisters and sinapisms. These are, in general, more advantageously applied to the lower extremities, than in the vicinity of the disease. Good effects have, nevertheless, been obtained from the application of a cataplasm wetted with muriatic acid to the anterior part of the larynx. All practitioners who have had occasion to see a good deal of this disease, will readily admit that these measures, although very rational and conformable to the results of experience in the treatment of inflammatory diseases in general, are nevertheless rarely sufficient, and that very few well-characterised cases have yielded to their influence. Others have consequently been had recourse to. I shall here notice those only which have been found decidedly beneficial. Of this kind are emetics, repeated daily, or even twice a day. They evidently accelerate the separation of the adventitious membrane, and favor its expulsion. However valuable this treatment may be,—and I have myself obtained cures which I could attribute to it alone,—it is no doubt too true that the greater number of cases still prove fatal, even when it is called in to aid the means already detailed. The internal use of hydro-sulphuret of potass was, some years since, cried up as a sort of specific in croup. It is one of the ancient family of the alkaline resolvents, by which the chemical physicians, followers of Sylvius of Leyden, proposed to correct the too great plasticity or viscidity of the fluids, and even to dissolve concretions already formed. I have before spoken of this alkaline treatment. In the present case it is sufficient to remark, that its effects are too slow to be of any use in a disease of such rapid progress as croup. In small quantity, its effects are insignificant; and, in larger and more repeated doses, it must be more injurious as an irritating, acrid, and almost caustic substance, than it can be beneficial as an alkaline remedy. Pretty numerous successful results have been obtained from mercurial frictions, exhibited in such doses as speedily to produce salivation; and, in the actual state of the science, I think no prudent practitioner ought to neglect this method, conjointly with blood-letting and emetics. The efficacy of this practice, even in a very striking degree, cannot be questioned in many other inflammatory diseases, particularly hepatitis and peritonitis. Still the success of even the mercurial treatment is not sufficiently great to hinder us from looking for other means; and if I had had occasion to treat this disease since I have experienced the efficacy of tartar emetic in large doses in many inflammatory diseases, I would certainly have had recourse to it with considerable confidence." (P. 125.)

The most remarkable circumstance in this extract is the assertion that the danger depends upon the mechanical effect of the false membrane, rather than the inflammation which gives rise to it; and, influenced by this opinion, the emetics are given to "favor its expulsion." Whereas, it is but too well known that the case will prove fatal if the inflammation be not subdued, even although the false membrane has been ejected. The manner in which mercury is spoken of in the form of friction, sufficiently shows that our neighbours either are ignorant of, or hesitate to adopt, the full and free doses of calomel which in this country have been found so efficacious.

*Bronchial Hemorrhage* is the term employed to designate the spitting of blood which proceeds from a simple exhalation from the surface of the mucous membrane. When tubercles burst into the bronchia, the slight bleeding which accompanies this accident arises from the rupture of small vessels, and hemorrhages of much greater extent follow the bursting of a vessel which traverses a tuberculous excavation; but it is now well ascertained that the greater number of cases of slight hæmoptysis consist in an exhalation from the bronchial membrane. In examining patients who have died during this disease, blood is found in the bronchia in greater or less quantity; the mucous membrane is generally softened, and stained with blood through its whole thickness. The discharge is small, and the blood frothy. The profuse hemorrhages, commonly called vomiting of blood, arise in most instances from pulmonary apoplexy, or that more severe disease originating in the vesicular structure of the lungs.

In bronchial hemorrhage, the chest is perfectly sonorous, and there is no crepitous, but only a mucous, rattle. The treatment recommended by our author—

"Consists most commonly in the more or less repeated use of blood-letting. Taking blood from the foot is generally preferable in women when there is suppression of the catamenia. When the detraction of much blood is not requisite, the application of leeches to the inner side of the thighs, or below the ankles, may be substituted. I give these places the preference to the vulva, for various reasons; and, among others, because I am convinced, from many comparative trials, that leeching from the last-mentioned place is not more effective as a derivative than from the other two, particularly if the blood is taken from both at the same time. Dry cupping or with lancets, sinapisms, stimulating foot-baths, may be usefully employed after venesection, or in cases where this is not necessary.

"Rest and absolute silence, a cool air, abstinence from wine and stimulant food, and a regimen proportioned in strictness to the severity and extent of the hemorrhage, are accessory measures by no means to be neglected. The same remark applies to the use of mucilaginous drinks, such as decoction of the roots of Grande Consonde or marsh mallow, rice water, solutions of gum arabic or tragacanth, &c. Acids and astringents are also commonly employed, particularly l'eau de Rabel, or the sulphuric acid sufficiently diluted, alum, tormentilla, bistort, kino, pomegranate bark, and, of late, rhatany root and its extract. These means are more hurtful than beneficial at the commencement of the hemorrhage; but they may sometimes be advantageously employed in old cases, which are combined with an atonic state of the system, or when the blood is only slightly coloured and feebly concrescible. In this last case I have sometimes used with benefit the dry sulphate of iron. To derive advantage from astringents, they must be given in larger doses than is usual. I give, for example, from one to four drachms of alum in a pint of any sweet mucilaginous drink.

"When an active hæmoptysis is checked, Sydenham advises the patient to be purged, and looks upon this means as most likely to prevent a relapse. I have always adopted the same practice, (except when evidently contra-indicated,) and, as it appeared, frequently with advantage. Obstinate spittings of blood, which have resisted repeated bleedings, sometimes terminate instantaneously under the effect of a purgative. (P. 129.)

***Polypus of the Bronchial Membrane*** is so rare a disease, that Laennec has only met with three examples of it on record. He mentions having seen a concretion in one of the large bronchia, which might easily have been mistaken for a polypus: although it almost plugged up the left branch, it neither impeded respiration on this side, nor prevented pectoriloquism in a cavity in the upper lobe.

***Ulcers of the Bronchia.*** This also is a rare affection, although our author thinks that it would be more frequently discovered if we were in the habit of examining the bronchia with greater care. The part of the membrane in which ulcers have been most frequently observed, is between the point where the trachea enters the thorax and its bifurcation. The size of the ulcers varies from a few lines to an inch and a half: they are of a grey colour, and covered with puriform mucus. The symptoms are—

"A pain, at first slight, or a simple feeling of irritation at the bottom of the trachea, experienced momentarily, and sometimes only when the patient sings, cries, or raises the voice in speaking. This state may continue a long time. I know a lady who has suffered in this way for ten years, without any other apparent alteration in her health. She has tried various means, and particularly the most powerful issues, without effect, and has only found relief in preserving absolute silence. After a time, the pain becomes constant, even when the patient is silent; and even then it is found that the voice is not always perceptibly altered in its character. Cough soon supervenes, attended by a colourless, ropy, pituitous expectoration, intermixed with opaque puriform particles. When this becomes abundant, a rattle, perceptible by the naked ear, is heard in the trachea; and, when not so heard, I have found it very distinct by means of the stethoscope. This instrument detected it at the same time in various parts of the lungs, while in many points it discovered the respiration to be very feeble. This was owing probably to the accumulation of phlegm in the smaller bronchia, since the respiration became good after expectoration. These symptoms are soon accompanied by extreme dyspnœa, the patient being obliged to remain in the sitting posture night and day; and, when he awakes after an imperfect sleep, he is apt to be seized with a suffocating cough, as if some foreign body had got into the trachea; and this continues until after the expectoration of a certain quantity of mucus. At this stage, emaciation, which had hitherto been slow in its progress, makes rapid advances, and sometimes produces extreme extenuation; and the patient at last dies with all the symptoms of the suffocative catarrh.

"Very great efforts of voice, acute cries, violent forcing of the head backwards, have sometimes appeared to be the occasional cause of the ulcers of the trachea. Cutaneous complaints and syphilis would seem also to predispose to them. Although sometimes met with in phthisical cases, they are found more commonly in subjects whose lungs are entirely sound. We must, however, except those cases where ulceration of the trachea or upper part of the bronchia is occasioned by the rupture of softened tubercles, situated in one of the cervical or bronchial glands. But ulcers of this kind cannot be considered as at all of an idiopathic nature, being entirely analogous to those fistulous openings produced by the discharge of a tubercle, abscess, or gangrenous eschar of the lungs, into the branches of the bronchia. Ulcers of this kind have a great tendency to cicatrisation, and are found after a certain time smooth, polished, and without any appearance of spreading. The tracheal ulcer, on the contrary, appears to have no tendency to cicatrise; and I know of no well authenticated instance of the cure of this affection." (P. 134.)

The treatment consists in the employment of local drains, particularly the repeated application of small moxas on the anterior and lower part of the neck; together with the preservation of absolute silence on the part of the patient.

The cartilaginous rings of the bronchia become occasionally ossified in old persons, but no perceptible alteration in the function of the lungs is connected with this change.

*Foreign Bodies in the Bronchia.*—

" Morsels of food, pins, needles, pieces of wood, stones of fruits, sometimes get into the bronchia. Violent irritation, convulsive cough, and (if the foreign body is large) threatening suffocation, are the immediate consequence of this accident, which is, nevertheless, unattended by any very immediate or pressing danger, unless the body introduced is sufficiently large to obstruct, more or less completely, the larynx or trachea. Cough, accompanied by a mucous or bloody expectoration, is the most usual symptom which supervenes to the accident; but even this soon passes off, particularly if the substance is small and falls down into a bronchial ramification: the organ becomes accustomed to the foreign body, and no inconvenience is produced by it.

" Accidents of this sort arise from very various causes. I was myself witness to a very singular case of the kind. Professor Corvisart, being desirous of exercising an unexpected supervision of some part of the clinical hospital, came to it one evening contrary to his custom, and suddenly entered the apartments of the steward, who had been indulging in a too plentiful repast. Taken by surprise, the man becomes sick at stomach, but, making a violent effort to repress vomiting, he falls to the ground and expires. On examining the body, the larynx, trachea, and bronchia are found filled with half-digested food.

" The ancient pathologists regarded foreign bodies introduced into the bronchia in a state of powder, as the cause of several severe diseases of those canals, as well as of the substance of the lungs; and, among others, of phthisis pulmonalis and the chalky concretions of the lungs, bronchial glands, or bronchial tubes. This opinion appears to me altogether without foundation. It is imagined that stonecutters and lapidaries are particularly subject to formations of this kind, occasioned by the inhalation of the dust amid which they work.

" It is needless to remark that this dust is entirely unlike the cretaceous formations in the lungs. On this subject it deserves notice that stage coachmen, who spend their life amid much more dust, are usually healthy, or suffer only from diseases produced by intemperance and the inclemency of the weather. It is indeed singular how little sensible the mucous membrane of the bronchia is to solid matters when reduced to an impalpable powder, when we know that the introduction of a body only a little larger, such as a bit of sugar, or even of a gummy or albuminous fluid, occasions extreme irritation and cough. Every one is occasionally caught in a cloud of dust, and merely experiences, while breathing in it, an oppression, without any inclination to cough. It is well known that, when we have been for some time breathing an air loaded with dust or smoke, these foreign bodies are, after a certain time, expectorated with the mucous secretion of the bronchia.

"For these reasons I consider the chalky formations in the bronchia, as well as every accidental production in the living body, as the result of perverted secretion. These productions in the bronchia I have only met with in branches which are dilated, or in the vicinity of old tuberculous excavations cured by the formation of a fistula or cartilaginous cicatrice; and we shall find hereafter, when treating of phthisis, that the development of cretaceous matter frequently succeeds that of tubercles." (P. 136.)

*Diseases of the Bronchial Glands.* These differ from all other lymphatic glands in their colour, which, in their centre at least, is a deep black, even in those whose lungs are perfectly sound. The colouring matter is united with the lymph, and, if a drop of it be applied to the skin, the stain is removed with difficulty. Inflammation of these glands appears to be very uncommon, and Laennec has seen very few cases in which they had run on to suppuration. Calcareous matter, however, and tubercles, are very common; and the glands sometimes attain the size of a hen's egg, several uniting into one mass. The tuberculous matter softening is either removed by absorption or by an opening into the bronchia; but the development of such tubercles is of little importance, provided the lungs are free from them. It is scarcely necessary to say that the natural black colour of the bronchial glands must not be confounded with Melanosis; a mistake which appears frequently to have occurred.

(To be continued.)

